# Augmentation of the Antibody Response of Atlantic Salmon by Oral Administration of Alginate-Encapsulated IPNV Antigens

**DOI:** 10.1371/journal.pone.0109337

**Published:** 2014-10-13

**Authors:** Lihan Chen, Goran Klaric, Simon Wadsworth, Suwan Jayasinghe, Tsun-Yung Kuo, Øystein Evensen, Stephen Mutoloki

**Affiliations:** 1 Department of Basic Sciences and Aquatic Medicine, Norwegian University of Life Sciences, Oslo, Norway; 2 EWOS Innovation AS, Sandnes, Norway; 3 Department of Mechanical Engineering, University College London, London, United Kingdom; 4 Department of Animal Science/Institute of Biotechnology, National Ilan University, Taipei, Taiwan; University of Liverpool, United Kingdom

## Abstract

The objective of the present study was to assess the effect of alginate-encapsulated infectious pancreatic necrosis virus antigens in inducing the immune response of Atlantic salmon as booster vaccines. One year after intraperitoneal injection with an oil-adjuvanted vaccine, post-smolts were orally boosted either by 1) alginate-encapsulated IPNV antigens (ENCAP); 2) soluble antigens (UNENCAP) or 3) untreated feed (control). This was done twice, seven weeks apart. Sampling was done twice, firstly at 7 weeks post 1^st^ oral boost and the 2^nd^, at 4 weeks after the 2^nd^ oral boost. Samples included serum, head kidney, spleen and hindgut. Serum antibodies were analyzed by ELISA while tissues were used to assess the expression of IgM, IgT, CD4, GATA3, FOXP3, TGF-β and IL-10 genes by quantitative PCR. Compared to controls, fish fed with ENCAP had a significant increase (p<0.04) in serum antibodies following the 1^st^ boost but not after the 2^nd^ boost. This coincided with significant up-regulation of CD4 and GATA3 genes. In contrast, serum antibodies in the UNENCAP group decreased both after the 1^st^ and 2^nd^ oral boosts. This was associated with significant up-regulation of FOXP3, TGF-β and IL-10 genes. The expression of IgT was not induced in the hindgut after the 1^st^ oral boost but was significantly up-regulated following the 2^nd^ one. CD4 and GATA3 mRNA expressions exhibited a similar pattern to IgT in the hindgut. IgM mRNA expression on the other hand was not differentially regulated at any of the times examined. Our findings suggest that 1) Parenteral prime with oil-adjuvanted vaccines followed by oral boost with ENCAP results in augmentation of the systemic immune response; 2) Symmetrical prime and boost (mucosal) with ENCAP results in augmentation of mucosal immune response and 3) Symmetrical priming and boosting (mucosal) with soluble antigens results in the induction of systemic immune tolerance.

## Introduction

Infectious pancreatic necrosis is an important disease of salmonids responsible for great economic losses in the aquaculture industry. It is characterized by loss of appetite, darkened skin pigmentation, distended abdomen and mortalities ranging from negligible to almost 100%. Histopathologically, necrosis of pancreatic acinar cells, multifocal hepatic necrosis and acute catarrhal enteritis are commonly observed [1], [2]. The causative agent is infectious pancreatic necrosis virus (IPNV), a double stranded RNA virus belonging to the family *Birnaviridae* and genus *Aquabirnavirus* where it is the type species.

Control of IPN is by vaccination and oil-based vaccines have earned their place in the market mainly because of their contribution to the control of bacterial diseases in the late 80s and early 90s in Norway. The efficacy of these vaccines against diseases caused by intracellular pathogens such as viruses however remains equivocal, thus the need for the continued search for more effective vaccines.

The most desirable vaccines for higher vertebrates and even more so for fish are those delivered orally because of the ease with which they are administered; are stress-free; applicable to smaller fish and are less labour-intensive [3]. Their usage in the aquaculture industry has however been under-exploited because of their poor performance in comparison with injectable and immersion counterparts. Some of the challenges associated with orally delivered vaccines include poor antigen delivery and uptake, degradation during passage through the digestive tract and induction of tolerance [4], [5]. Nevertheless, a report of good protection in fish vaccinated with encapsulated DNA plasmids has recently been published [6]. Unfortunately, legislation in most countries at the moment precludes the use of DNA vaccines in food animals [7], [8].

One of the challenges faced by vaccination of fish is the duration of protection conferred by different preparations. As already mentioned, oil-based vaccines induce long lasting protection against several bacterial pathogens but this could be at the cost of severe side effects [9]. For viral diseases including IPN, most products on the market do not give satisfactory protection probably because of their failure to induce sufficiently high antibody titers required prior to challenge [10]. Boosting is a good alternative for enhancing or extending protection as shown for lactococcosis [11]. The effect of boosting against IPNV in particular and oral vaccination in general is however not well understood. The main purpose of the present study therefore was to assess the effect of alginate-encapsulated IPNV in stimulating the immune system of Atlantic salmon as a booster vaccine.

## Results

### Intake of oral boost feeds and IPNV antigen dose

The average weight of the fish during the primary and secondary oral boost feeding, the feed intake and antigen dose are shown in Table 1. As targeted, the average antigen dose administered during each of the boost periods was about 1×10^9^ TCID_50_/fish. However, due to the doubling in the fish weight between the two boost periods, the dose per kg of fish body weight during the second boosting was almost half that during the first (Table 1).

**Table 1:** Fish size in unit of mass (g), Weekly feed intake (FI) per fish, weekly IPNV antigen dose per fish and weekly IPNV antigen (Ag) dose per unit of fish mass (dose/kg).

Period	Group	Feed	Fish size (g) ±SD	Feed intake (g/fish/week) ±SD	IPNV Ag dose (TCID50/fish/week) ±SD	IPNV Ag dose (TCID50/kg/week) ±SD
	Control	CF-1	395±92	24.7±0.4	0.00	0.00
Primary boost	Unencap	OBF-1	375±82	23.9±2.4	9.6×10^8^±1×10^8^	2.6×10^9^±6×10^8^
	Encap	OBF-2	426±121	23.1±0.9	9.3×10^8^±4×10^7^	2.2×10^9^±6×10^8^
	Control	CF-2	846±135	39.6±0.4	0.00	0.00
Second boost	Unencap	OBF-4	796±126	37.3±3.9	1.1×10^9^±1×10^8^	1.4×10^9^±3×10^8^
	Encap	OBF-5	782±105	38.0±1.3	1.1×10^9^±4×10^7^	1.5×10^9^±2×10^8^

### Antigen retention in head kidneys and hindguts

To estimate the amount of antigen taken up and retained both locally and systemically in each group, qPCR was used targeting hindgut and head kidneys tissues to examine retained antigens at the time of sampling (7 weeks and 4 weeks following the 1^st^ and 2^nd^ boosts, respectively). The head kidney was used to represent the systemic compartment since this is one of the main antigen trapping organ for blood-borne antigens in fish [12].

**Figure 1:**
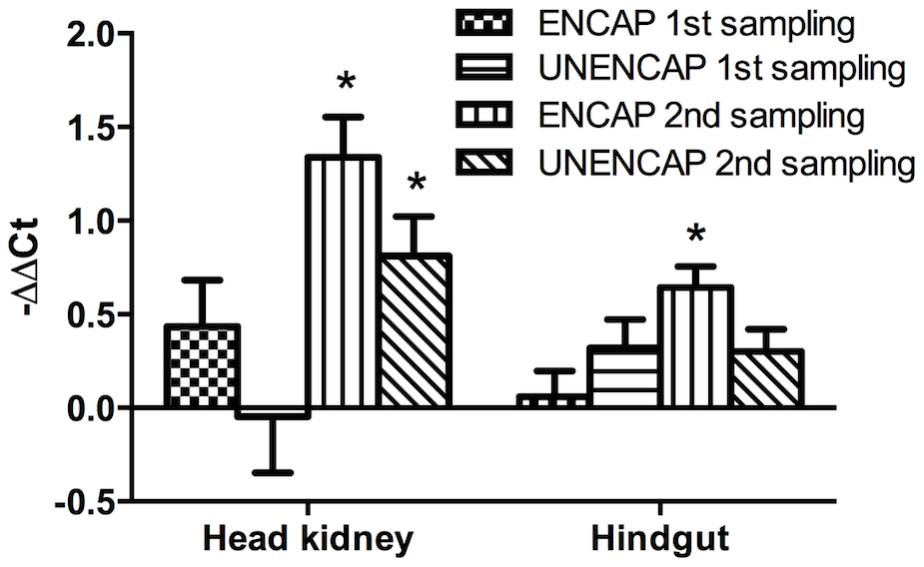
Infectious pancreatic necrosis virus (IPNV) mRNA expressed by real time RT-PCR in selected organs of Atlantic salmon following oral boost with different antigen preparations. This assay was used as a surrogate marker of retained formalin-inactivated IPNV antigens in the present study. All fish were vaccinated with an oil-based vaccine one year prior to the start of this study. The control fish received no booster oral antigens. n = 30; *statistically significant p<0.05.

The results show that more mRNA of IPNV (used as a surrogate of antigens) were retained in the encapsulated (ENCAP) versus unencapsulated (UNENCAP) groups at both time points (Fig. 1). In the head kidney, a significant increase in antigens retained from the 1^st^ to the 2^nd^ sampling was observed in both groups. A similar trend in the ENCAP group was observed in hindgut while for the UNENCAP group, no difference between sampling times was observed (Fig. 1).

### Oral boosting with alginate-based antigens induces a systemic but transient IgM antibody response

Since all experimental fish had previously been vaccinated with an oil-based vaccine that contained IPNV antigens, all the fish had relatively high specific background antibodies as expected. The un-boosted group (control) was used as the baseline for comparison with boosted groups.

**Figure 2:**
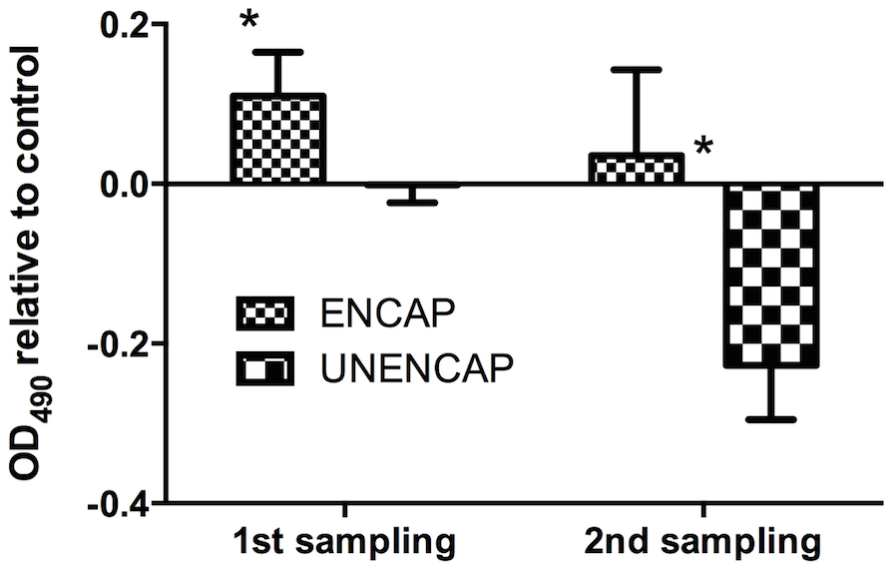
Infectious pancreatic necrosis virus-specific serum antibody levels in orally boosted groups of Atlantic salmon relative to un-boosted controls. Antibodies were assessed by ELISA and the results were obtained by using a plate reader (TECAN, Genios) at a wavelength of 492 nm. n = 30; *statistically significant p<0.05.

In general, the response of antibodies in boosted groups showed a reverse trend over time compared to that of antigens (Fig. 1&2). In the group boosted with ENCAP, the antibody response was significantly higher (p<0.04) than the control following the 1^st^ boost (Fig. 2). At 4 weeks following the 2^nd^ boost however, the antibodies had returned to background levels. In the UNENCAP group, no difference was observed compared to unboosted controls during the 1^st^ sampling while the antibodies were significantly suppressed (p<0.02) following the 2^nd^ boost (Fig. 2).

### The systemic immune response is predominantly Th2

The induction of antibodies in the ENCAP group was suggestive of a predominantly humoral response. Thus to verify this, we examined the expression of CD4 and GATA-3 genes that are known to be associated with Th2 responses, in the head kidney and spleen.

**Figure 3:**
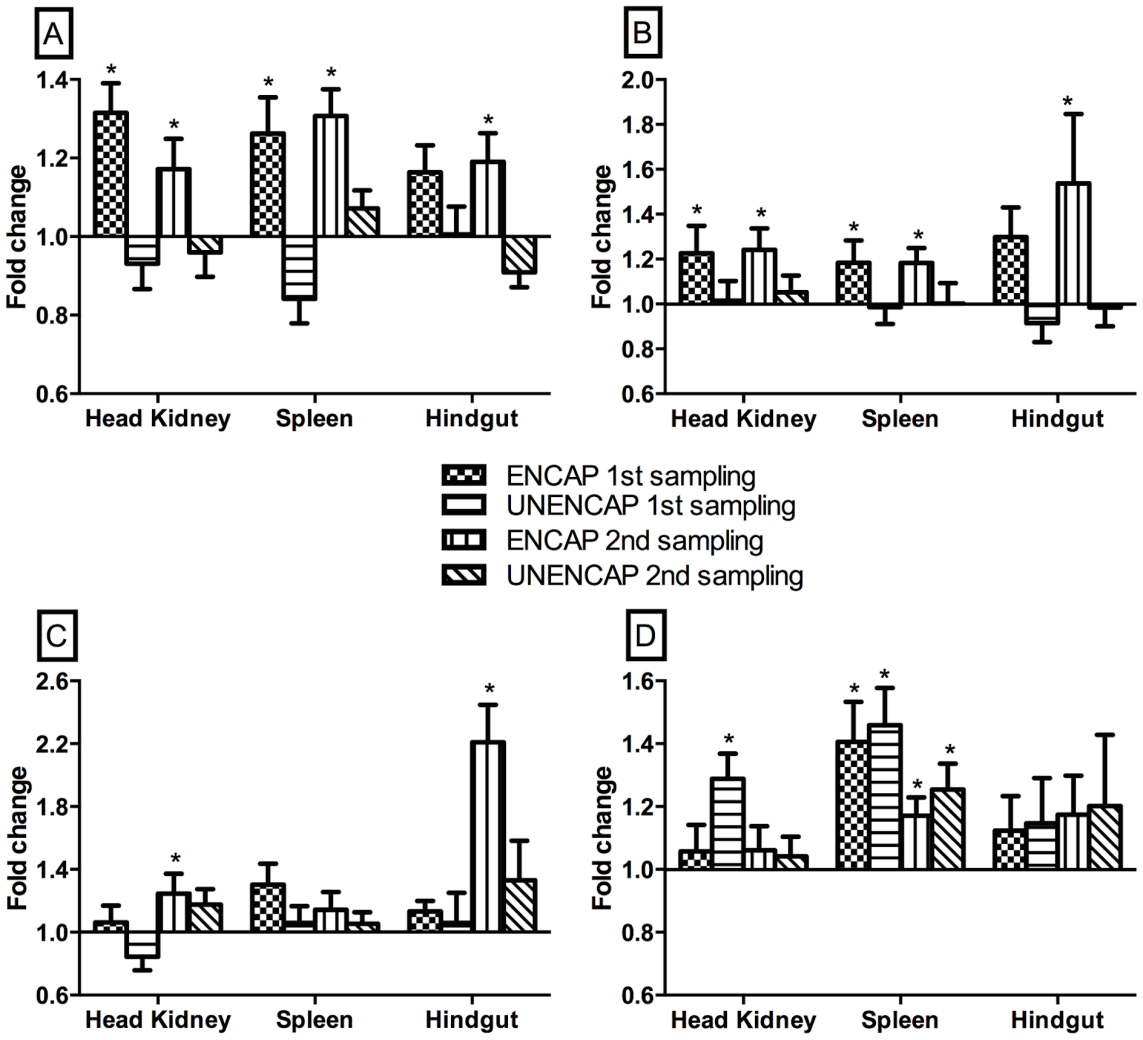
Mean relative expression of A) CD4; B) GATA3; C) IgT; and D) IgM genes of fish orally boosted with different antigen preparations compared to un-boosted controls. Real-time RT-PCR. n = 30; *statistically significant p<0.05.

At both 7 weeks post primary- and 4 weeks post-secondary boost, the ENCAP group had significantly higher CD4 expression (p<0.04) in both the head kidney and spleen compared to other groups (Fig. 3a). In contrast, this gene was suppressed in the UNENCAP group at the 1^st^ sampling albeit non-significantly. At the 2^nd^ sampling, this gene was significantly suppressed (p<0.01) in this group. In the ENCAP group, the expression of GATA3 was significantly up-regulated (p<0.04) at both time points in the head kidney and spleen, consistent with the results of CD4 while in the UNENCAP group, these genes were not induced (Fig. 3b).

### The gut mucosal response is also Th2 and is primarily primed by the oral route

To assess the gut mucosal response, we examined the expression of IgT mRNA since antibodies are not available to us. In addition, mRNA expression of IgM, CD4 and GATA3 genes were also assessed.

After the 1^st^ oral boost, marginal but non-significant inductions of IgT were observed in both the ENCAP and UNENCAP groups compared to the control (Fig 3c). At 4 weeks following the 2^nd^ boost, the expression increased in both groups but more significantly (p<0.01) in the ENCAP compared to UNENCAP group. Interestingly, CD4 and GATA3 expression in this organ had a similar pattern to IgT (Fig. 3a&b), especially for the ENCAP group. Conversely, CD4 and GATA3 expression of the UNENCAP group were generally not induced at both time points. The expression of IgM in the hindgut was not differentially expressed in all groups at all-time points examined (Fig. 3d).

The assessment of IgT expression was extended to the head kidney and spleen where at all-time points and in all groups, this gene was not differentially expressed. The only exception was in the head kidney of the ENCAP group, at 4 weeks following 2^nd^ boost (Fig. 3c), where it was significantly up-regulated (p<0.03).

### Repeated oral administration of IPNV antigens results in decreased serum antibodies but not in the hindgut of Atlantic salmon

The reduction in serum antibodies following administration of antigens can be due to consumption [10], [13]. Thus in order to check whether this was the case in the present study, we examined the transcript levels of IgM. Fig. 3d shows significant down-regulation of B cell IgM transcripts both in the kidneys (p<0.01) and spleen (0.001) of the UNENCAP group from the 1^st^ to the 2nd samplings. A similar change was observed in the spleen of the ENCAP group (p<0.01) but without any differential regulation in the kidney.

In order to gain insight into the suppression of antibody production by B cells in the UNENCAP group, we examined the expression of forkhead box protein 3 (FOXP3), TGF-β and IL-10.

**Figure 4:**
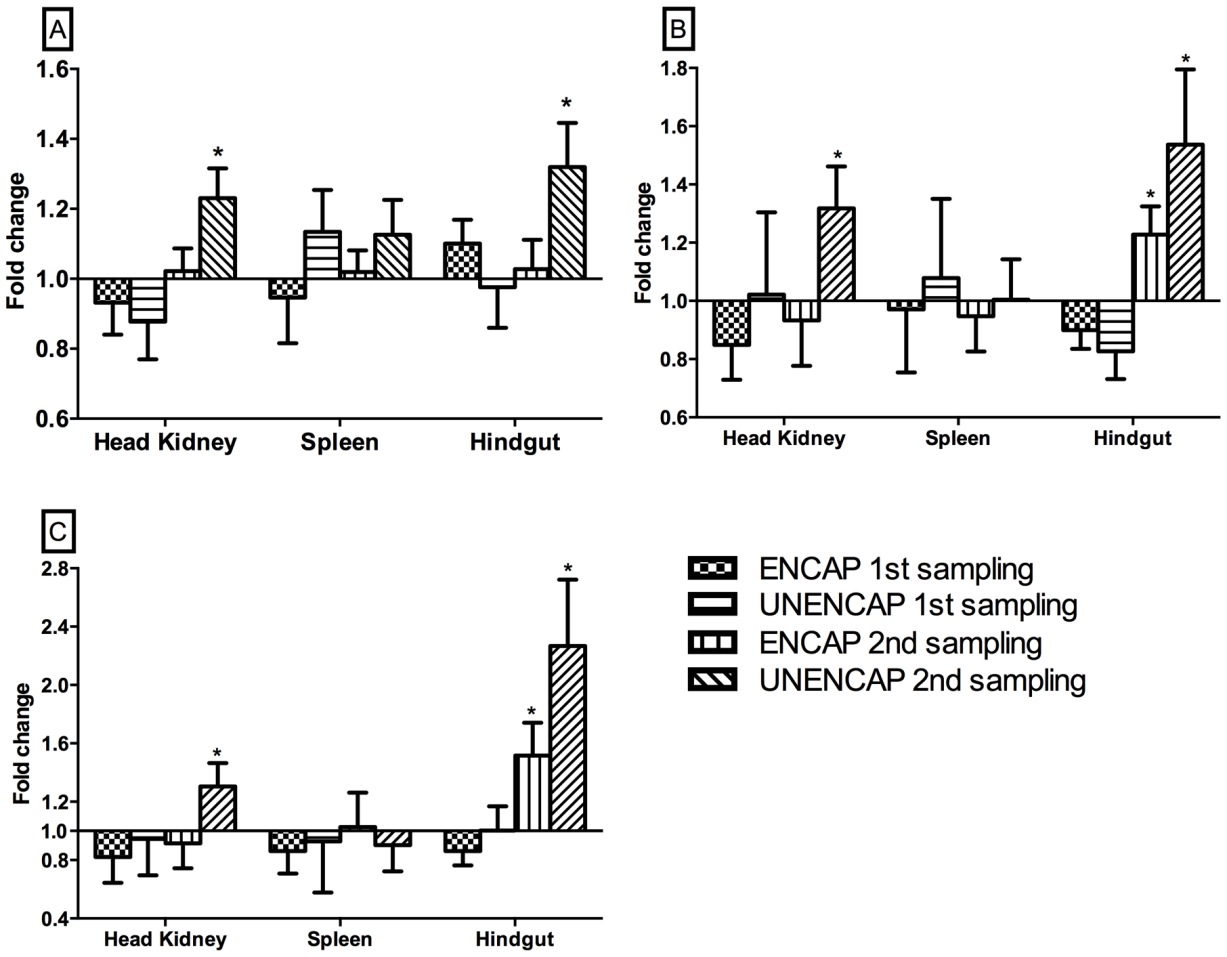
Mean relative expression of A) FOXP3; B) TGF-β and C) IL-10 genes in fish orally boosted with different antigen preparations compared to un-boosted controls. Real-time RT-PCR. n = 30; *statistically significant p<0.05.

Consistent with the reduction/suppression of antibodies in the UNENCAP group at 4 weeks following 2^nd^ boost, the expression of FOXP3, TGF-β and IL-10 were all significantly up-regulated (p<0.003, 0.035 and 0.0048, respectively) in the kidneys of this group (Fig. 4a–c). In contrast, no differential expressions of either of these genes were observed in any of the groups after the 1^st^ boost or in the spleen.

In the hindgut, FOXP3 was only induced in the UNENCAP group after the 2^nd^ boost (Fig. 4a) while both TGF-β and IL-10 were significantly induced in both ENCAP and UNENCAP groups (p<0.02 and 0.01 for TGF-β and p<0.01 and 0.01 for IL-10, respectively).

## Discussion

The findings of the present study demonstrate that oral boosting of Atlantic salmon following parenteral injection with alginate encapsulated IPNV antigens induces both systemic and mucosal (gut) humoral responses. The significant up-regulation of CD4 (p<0.02) and GATA-3 (p<0.04) genes in the head kidneys and spleens of the ENCAP group (Fig. 3) fit very well with the induction of serum antibodies and point towards a predominantly T-helper 2 (T_H_2) response. In higher vertebrates, the intestinal mucosa contains high basal levels of IL-4, IL-10 and TGFβ that are induced shortly after oral vaccination [14]. This micro-environment is thought to tip responses of oral vaccines towards T_H_2 [4]. Furthermore, the main mechanism of action of alginate encapsulated antigens has been proposed to be biased towards T_H_2 responses [15]–[18]. These findings are consistent with reports of others using different antigens [19], [20] and suggest that oral boosting with alginate encapsulated antigens holds promise as a means of augmenting immune responses against IPN. One limitation of this study is that the fish were not challenged following vaccination and therefore the protective effects of the responses could not be tested but should be a subject for future studies.

There are conflicting reports when it comes to protection against disease using orally administered vaccines, with some reporting success [6], [11], [20] while others found little or no difference from controls [17]. Several reasons can be attributed to these variations including the nature of antigens used, formulation of oral preparations, immune response generated versus that desired etc. In the present study, the fish were not challenged following vaccination owing to logistical constraints. While this should be a subject for further studies, it is known from a previous study that high antibody titers against IPNV at the onset of challenge correlate with protection of the fish [10]. It is not unlikely therefore that the augmentation of the immune response in the present study may have been associated with protection.

It has previously been reported that oral vaccination results in transient antibody response lasting typically 3 weeks post vaccination [20]–[22], and most of the studies address parenteral/oral combinations or vice-versa. However, very few studies have examined the effect of repeated oral vaccination. In the present study, reductions in serum antibodies following two oral vaccination (7 weeks apart) was observed in the both the ENCAP and UNENCAP groups after the second oral boost. This is consistent with the findings of others who used a similar administration regime (5 days of oral vaccine administration per month) [17]. In the same study however, administering the oral vaccine at a rate of 3 days/week for 2 months resulted in progressive increase in antibody titers over time. Together, these findings suggest that modality by which oral vaccines are administered can determine whether a booster effect or tolerance ensues. The low CD4 expression in the UNENCAP group in the kidney and also its decline in the ENCAP group concurrent with the lack of induction of GATA3 and IgM expression in the present study is however intriguing. It is tempting to speculate that this could point towards anergy as discussed further below.

The induction of the systemic response as measured from serum antibodies and immune gene expression in the kidney of the ENCAP group following the 1^st^ oral boost is in line with previous reports [20], [22]. The fact the 1^st^ oral boost did not induce a corresponding change in the mucosal response as measured by gene expression in the hindgut suggests that injection vaccination does not activate mucosal immunity, in common with findings of others [20]. Furthermore, these results (Fig. 3a–c) suggest that in this study, the 1^st^ oral boost served as a “prime” to the mucosal response while the 2^nd^ one “boosted” it. Interestingly, oral boosting had no effect on the IgM expression in the hindgut, a difference from what others have observed assessing mucosal antibodies [20]. The ability of orally administered antigens to stimulate both systemic and mucosal immune responses on one hand and parenteral vaccination inducing only a systemic response demonstrate asymmetrical responses of immune induction as previously observed in mice [23].

One of the challenges of oral vaccination in fish is the induction of tolerance. This has been shown to be easily induced with soluble antigens [21]. In the present study, tolerance was induced by two booster administration of encapsulated IPNV antigen (UNENCAP) feeds (Fig. 2). In higher vertebrates, tolerance is the default immune pathway in mucosal surfaces and is related to the dose of antigens given, i.e. high doses lead to anergy/deletion while the opposite leads to regulatory T cells (Treg) induction [24]. The suppression of antibodies in the present study coupled with the induction of FoxP3, TGF-β and IL-10 (Fig. 4) suggests the involvement of both mechanisms. In higher vertebrates, FOXP3 is a key transcription factor of regulatory T cells (Tregs) while TGF-β is known to induce T cells including Tregs [24], [25]. IL-10 on the other hand is an anti-inflammatory cytokine that has been shown to contribute towards the induction of immune tolerance [24]. These genes have also been described in fish although their functions relative to immune tolerance remain to be characterized.

While the induction of tolerance may be testimony that much of the un-encapsulated IPNV antigens were taken up, meaning they survived the hostile acidic environment in the stomach, this finding underlines the importance of encapsulation as an aid to stimulating the immune response of fish. It is noteworthy that the doses of vaccines administered during the 2^nd^ boost were lower per body weight of fish compared to the 1^st^ boost since the fish had gained weight by the time they received the second boost. The effect of this was not addressed but should be a subject of future studies.

Finally, the findings of the present study can be summarized as follows: 1) Parenteral prime with oil-adjuvanted vaccine followed by oral boost with ENCAP results in a the augmentation of both the systemic and mucosal immune responses; 2) Mucosal (gut) immunity is primarily primed by oral administration of antigens; 3) Oral prime and boost with ENCAP results in transient augmentation of mucosal immune responses and 4) Oral priming and boosting with UNENCAP results in the induction of tolerance.

## Materials and Methods

This study was approved by the Norwegian Animal Research Authority. Prior to sampling, the fish was anaesthetised with Finquel (Scanvacc) at 100 mg/L in order to prevent suffering.

### Cell culture

Asian grouper strain K (AGK) cells and Chinook salmon embryo cells (CHSE-214; ATCC CRL-1681) [26] were maintained with L-15 medium (Invitrogen) supplemented with 10% L-glutamine and 1 µl/ml of gentamicin. In addition the medium used with the former also contained 7.5% fetal bovine serum (FBS) and these cells were kept at 28°C while the medium for CHSE cells contained 10% FBS and the cells were maintained at 20°C.

### Fish

The experiment was conducted at EWOS Innovation AS facilities in Dirdal, Norway. Healthy Atlantic salmon growers reared in sea water were used. The fish had been vaccinated with ALPHA JECT micro 6 (PHARMAQ) about a year prior to the first boost treatment and were kept at a water temperature of 12°C throughout the experimental duration.

### Vaccine preparations

#### Antigen preparation

A recombinant Sp strain of IPNV (rNVI-15PTA) [10] was used and was prepared as reported previously (Chen et al., 2013). Briefly, approximately 80% confluent AGK cells maintained in L15 media as described above but with 2% FBS were inoculated with IPNV using MOI = 0.1 followed by incubation at 15°C. Note: CHSE or RTG-2 cells can be used as alternatives in the absence of AGK cells, with post-culturing concentration to increase virus amounts if necessary. The virus was harvested following full CPE by centrifugation of the suspension at 2500×g followed by recovery of the supernatant. Titration of the virus was by end point dilution and the titer measured using the Spearman–Karber's 50% tissue culture infectious dose (TCID_50_) in CHSE-214 cells.

The virus was inactivated with formalin (0.5% final concentration equal to 0.2% formaldehyde) at room temperature for 48 hours with continuous stirring using a magnetic stirrer. Thereafter formalin was removed by dialysis. Inactivation was confirmed by inoculating confluent CHSE-214 cells while formalin residual effects were tested by incubating cells with excessive inactivated virus and assessing for toxicity.

#### Antigen encapsulation and feed preparation

The treatment groups of this study comprised either of the following feeding regimes: 1) untreated feed (control); 2) feed containing unencapsulated IPNV (in suspension); 3) feed containing alginate-encapsulated IPNV antigens.

Oral boost feeds (OBFs) were prepared by applying an oil mixture (OM) to Ewos Opal 200 base pellet (BP) in a vacuum infusion coating process. OMs were formulated by mixing IPNV Ag suspension (10^9^ TCID_50_/g), phosphate buffered saline (PBS), (2.70×10^10^ TCID_50_/g) with fish oil. Mixing was performed by using a high-performance disperser (Model T25, IKA Werke GmbH & Co., Germany) at ambient temperature. The OBF were composed with the aim of generating feeds with an antigen level of 4.01×10^7^ TCID_50_/g. This level was selected due to an expected daily feed intake of 3.56 g/fish during the first oral boost period. For the same reason, the targeted antigen level in the second OBF was 2.99×10^7^ TCID_50_/g. Control feeds were produced by mixing PBS with fish oil in advance of applying to BP in the vacuum infusion coating process.

### Trial design and oral boosting

**Figure 5:**
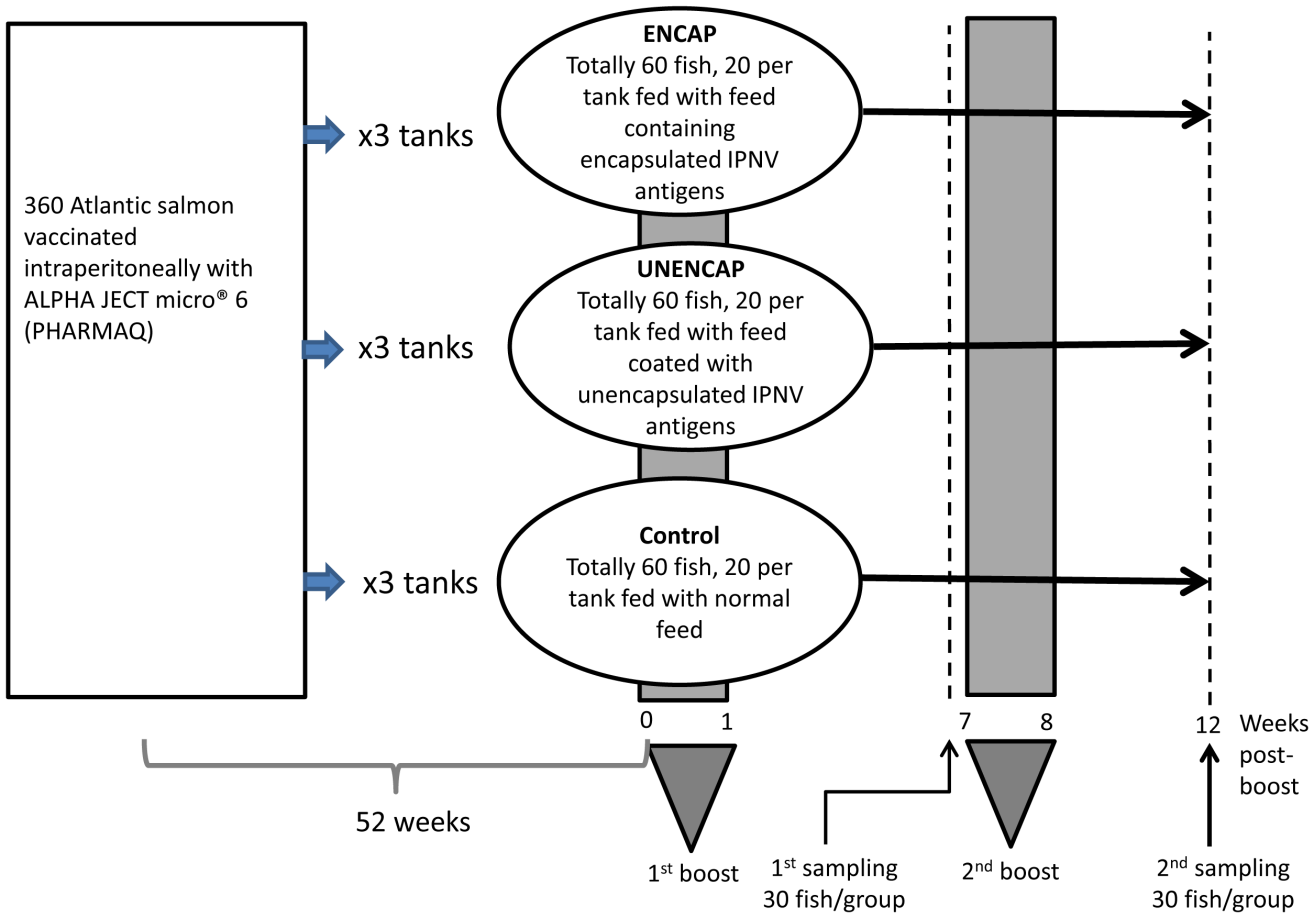
Schematic illustration of the trial plan used in the present study. Atlantic salmon growers previously (1 year) vaccinated against IPNV were split into three groups. Each group was further divided into three replicates (tanks) that were boosted twice orally for one week. Sampling was at 7 weeks post primary boost (a day before the second boost) and at the end of the trial. Ten fish from each tank (30 fish per group) were sampled at each time point.

As part of a larger study examining responses of Atlantic salmon to different alginate formulations, 360 healthy Atlantic salmon weighing approximately 200 g each were distributed by dip netting and sequential allocation into 9 circular 500 L tanks containing sea water 10 weeks prior to the start of the primary oral boost. A description of the fish is given in section 4.2. The tanks were randomly divided into three groups (Unencap, Encap, and Control), with three tanks being assigned to each group (Figure 5).

The fish were fed with Ewos Opal 200 diet for 10 weeks prior to the first boost and also until the second boost. All fish groups were fed ad-libitum. During primary boosting, normal feed was replaced by OBFs for 7 days. After 7 weeks, the fish were sampled. Sampling was performed on 10 fish from each tank by first anaesthetizing the fish with Finquel (Argent Laboratories) at 100 mg/L. The following samples were collected, blood in heparin tubes for serum extraction; head kidney, spleen and hind gut in both RNAlater (Invitrogen) and formalin.

Following sampling, a secondary boost was performed as described above. From then on, the fish were fed with Ewos Opal 500. The second sampling was done 4 weeks after the secondary boost.

### Feed intake assessment

Unconsumed feed was collected during both boost periods to calculate feed intake. Uneaten pellets were spilled out of the tanks within 10 min post feeding and filtered off from the outlet water using an automatic collection system. Residual pellets were removed from the filters and put into a drying cabinet for 24 h at 70°C. Amount of feed consumed was calculated as the difference between the dry weight of the feed served and the dry weight of unconsumed feed, expressed as the mass of feed per week per fish.

### Enzyme-linked immunosorbent assay (ELISA)

Blood samples were centrifuged at 2500×g for 10 min immediately after sampling. Thereafter, the serum was aspirated and transferred to new tubes that was the kept at -80°C until required.

ELISA was done as previously described [26] with minor modifications. Briefly, the wells of ELISA plates (Immunoplates, Nunc Maxisorb, Denmark) were coated with 100 µl of polyclonal anti-IPNV [27] diluted 1∶2000 in coating buffer (0.1 M Carbonate buffer pH 9.6) and then incubated at 4°C overnight. The plates were washed prior to the incubation of 200 µl of 5% dry milk per well for 2 hrs at room temperature. All washing steps were done in triplicate with 200 µl PBST/well, all dilutions were with 1% dry milk and all incubation was at room temperatures unless otherwise stated. After washing, the wells were incubated with 100 µl of IPNV supernatant (10^8^ TCID_50_/ml) for 2 hours. Following another washing step, serum samples diluted 1∶40 were then added to the wells and then incubated at 4°C overnight. After washing, 100 µl of mouse antibody against rainbow trout IgM [28] diluted in 1∶5000 was incubated for 2 hours. Following another wash, 100 µl of a 1∶1000 dilution of peroxydase conjugated anti-mouse Ig (DAKO, Denmark) was incubated in each well for 1 hour. 100 µl of OPD substrate (O-phenylenediamine dihydrochloride, DAKO) diluted in water was added to each well after washing. This reaction was incubated for 15 min following which the reaction was stopped by the addition of 50 µl/well 1 M H_2_SO_4_. Results were analyzed by using an ELISA reader (TECAN, Genios) at 492 nm.

### RNA isolation and quantitative real-time RT-PCR

Total RNA was isolated by using the RNeasy Plus minikit (Qiagen) according to the manufacturer's instructions, and the concentration of RNA was determined by using the Nanodrop ND1000 (NanoDrop Technologies).

Quantitative PCR was performed by using QuantiFast SYBR Green RT-PCR Kit (Qiagen) and the LightCycler 480 system (Roche). For each gene, 50 ng of RNA was used as a template in a mixture of specific primers (250 µM) (Table 2) and QuantiFast SYBR Green RT-PCR master mix in a total volume of 20 µl. The mixtures were first incubated at 50°C for 10 min, then 95°C for 5 min, followed by 40 amplification cycles (10 s at 95°C; 30 s at 60°C and 8 s at 72°C). The sequences of primers used are given in Table 2.

**Table 2:** Sequences of primers used in this study.

Genes	Accession number	Primer sequence 5′-3′	D*
β-actin	BT047241.2	CCAGTCCTGCTCACTGAGGC	F
		GGTCTCAAACATGATCTGGGTCA	R
IgT	HQ379938.1	AGAGGTGAAGACACACCGGTCATT	F
		ACGGAGTAGTTGCCTTTCTGGGTT	R
CD4	DQ867019.1	GAGTACACCTGCGCTGTGGAAT	F
		GGTTGACCTCCTGACCTACAAAGG	R
GATA3	NM001171800.1	CCCAAGCGACGACTGTCT	F
		TCGTTTGACAGTTTGCACATGATG	R
IgM	AF228580.1	TGAGGAGAACTGTGGGCTACACT	F
		TGTTAATGACCACTGAATGTGCAT	R
FOXP3	HQ270469	AGCTGGCACAGCAGGAGTAT	F
		CGGGACAAGATCTGGGAGTA	R
TGF-β	BT059581.1	AGTTGCCTTGTGATTGTGGGA	F
		CTCTTCAGTAGTGGTTTGTCG	R
IL-10	AB118099.1	CGCTATGGACAGCATCCT	F
		AAGTGGTTGTTCTGCGTT	R
IPNV	NC_001915.1	ATGCCAAGATGATCCTGTCCCACA	F
		TGCCTTTGAGGTTGGTAGGTCACT	R

D* direction of primer.

The 2^−ΔΔCT^ method was used to calculate the gene products as described elsewhere [29] and is the relative mRNA expression representing the fold induction over the control group. All quantifications were normalized to β-actin.

### Statistical analysis

The amount of feed intake for the three groups, gene expression and Elisa results were analyzed using Student's t test. F test was used to determine if the variances of population were equal or not. The threshold for significance was p<0.05 for both Student's t test and F test.
